# Role of osteocalcin, tumor necrosis factor-alpha and adiponectin in polycystic ovary syndrome patients with insulin resistance

**DOI:** 10.4274/tjod.61224

**Published:** 2017-06-15

**Authors:** Gönül Erkan, Ahter Tanay Tayyar, Gökhan Açmaz, İptisam İpek Müderris, Gülden Başkol, Fahri Bayram

**Affiliations:** 1 Private Hüma Hospital, Clinic of Obstetrics and Gynecology, Kayseri, Turkey; 2 Zeynep Kamil Maternity and Children’s Diseases Training and Research Hospital, Clinic of Obstetrics and Gynecology, İstanbul, Turkey; 3 Kayseri Training and Research Hospital, Clinic of Obstetrics and Gynecology, Kayseri, Turkey; 4 Erciyes University Faculty of Medicine, Department of Obstetrics and Gynecology, Kayseri, Turkey; 5 Erciyes University Faculty of Medicine, Department of Biochemistry, Kayseri, Turkey; 6 Erciyes University Faculty of Medicine, Department of Clinical Endocrinology and Metabolism, Kayseri, Turkey

**Keywords:** Polycystic ovary syndrome, insulin resistance, osteocalcin, Adiponectin, tumor necrosis factor-alpha

## Abstract

**Objective:**

Insulin resistance (IR) seems to be the main pathogenic factor in polycystic ovary syndrome (PCOS). Adiponectin and tumor necrosis factor-alpha (TNF-α) are important in IR. The aim of this study was to evaluate the correlations of osteocalcin, adiponectin, and TNF-α with IR in PCOS.

**Materials and Methods:**

A total of 60 women were divided into two groups. The first group constituted 44 patients with PCOS and the control group comprised 16 healthy women. Osteocalcin, adiponectin, TNF-α levels, body mass index (BMI), and IR in the fasting state were assessed and correlations of these parameters were evaluated.

**Results:**

Homeostasis model assessment (HOMA)-IR, adiponectin, osteocalcin, and androstenedione levels were significantly increased in the PCOS group. A moderate positive correlation between BMI and HOMA-IR, a moderate negative correlation between TNF-α and osteocalcin, and a mild negative correlation between adiponectin and BMI were detected in PCOS.

**Conclusion:**

Osteocalcin may have impact on adiponectin, TNF-α, and IR levels in PCOS. Different osteocalcin levels in patients with PCOS may be responsible for explaining PCOS heterogeneity.

## PRECIS:

Bone is recognized as an endocrine organ. Osteocalcin seems to play a key role in the heterogeneity of polycystic ovary syndrome.

## INTRODUCTION

Polycystic ovary syndrome (PCOS) is a common and heterogeneous disease characterized by anovulation, hyperandrogenism, and/or polycystic ovaries^(1,2)^. Therefore, an important consideration is whether such adipocytokines as adiponectin, a potential mediator of insulin resistance (IR), are also implicated in the pathogenesis of PCOS^(3)^. Levels of adiponectin, an abundant adipocyte-derived cytokine, are strongly correlated with measures of IR^(4,5)^. Gonzalez et al.^(6)^ illustrated that hyperglycemia caused an increase in reactive oxygen species (ROS) generation from peripheral blood mononuclear cells (MNC). ROS-induced oxidative stress is a known activator of nuclear factor B, a proinflammatory transcription factor that promotes tumor necrosis factor (TNF) gene transcription^(7)^. TNF was established as a mediator of IR by Hotamisligil et al.^(8)^. Thus, increased TNF release from MNC in response to hyperglycemia may be an underlying mechanism for IR in PCOS.

Previous animal studies showed that osteocalcin stimulated the expression of insulin in islets and of adiponectin in adipocytes with increased insulin secretion and sensitivity^(9)^. Reduced osteocalcin levels have been claimed to be associated with diabetes mellitus (DM) development^(10)^. We aimed to evaluate the correlations of blood osteocalcin, adiponectin, and TNF-α levels with IR in PCOS. Additionally, we evaluated the relationship of these with some hormonal parameters.

## MATERIALS AND METHODS

A total of 60 women including 44 patients with PCOS and 16 healthy women (control group) were studied at Erciyes University Gynecology Clinic. The diagnosis of PCOS was based on the established guidelines by the PCOS Consensus Workshop Group^(1)^. Ultrasonographic diagnosis of polycystic ovaries was based on the presence of 12 or more follicles in each ovary measuring 2-9 mm in diameter, and/or increased ovarian volume >10 mL on pelvic or vaginal ultrasound examination. Oligomenorrhea was defined as the absence of menstruation for 35 days or more and amenorrhea was defined as the absence of menstruation for 3 months or more^(1)^.

All women were examined both clinically and gynecologically including ultrasonography. Body weight, height, and body mass index (BMI) were recorded. The BMI was calculated as weight/(height)^2^ in kilograms per square meter. The study and control groups were weight matched. Patients with congenital adrenal hyperplasia, androgen-producing tumors, adrenal dysfunction, Cushing’s syndrome, hyperprolactinemia, DM, liver, kidney, heart, and thyroid diseases were excluded from the study. None of the women in study or control group had taken medications known to effect plasma sex steroids for ≥6 months before the study and none of the volunteers was a cigarette smoker. All the women agreed to participate in the present study. The study was approved by the Ethics Committee of Erciyes University Hospital (approval number: 2011-369) and written informed consent was obtained from each woman. Moreover, we obtained an Australian-New Zealand clinical trials registry number: 12613001132730.

Fasting state venous blood was collected from the subjects during the midfollicular phase of the menstrual cycle between 08:00 am and 09:00 am. Glucose levels were measured three days after the normal diet and normal daily activity using the oxidase method with Konelab 60-i auto-analyzers (Thermo Clinical Labsystem, Finland). IR in the fasting state was assessed by using homeostasis model assessment (HOMA) and was calculated with the following formula: fasting plasma glucose (mmol/L) × fasting serum insulin (µU/mL) divided by 22.5, as described by Matthews et al.(11). Hormonal analyses included: thyroid stimulating hormone, dehydroepiandrosterone sulfate (DHEAS), prolactin (PRL), luteinizing hormone (LH), follicle-stimulating hormone (FSH), estradiol (E2), 17-hydroxyprogesterone (17-OHP), androstenedione (A), free testosterone (fT), total testosterone (tT), insulin, and sex hormone binding globulin (SHBG) levels. tT and fT (Biosource, Nivelles, Belgium), 17-OHP (DSL-3500, Texas, USA), DHEAS (Immunotech, Marseille, France), A (DSL-3800, Texas, USA) were measured using an immunoradiometric assay and its commercial kit, serum SHBG (Zentech, Angleur, Belgium), insulin (Biosource, Nivelles, Belgium), LH, FSH, P, PRL (ACS:180, Bayer, Germany) were measured using chemiluminescence and a commercial kit. After centrifugation, blood serum was stored at -70 °C until assayed. Adiponectin (Adiponectin kit, Assaypro, UK), TNF-α (TNF-α Invitrogen 96 Tests, UK) and osteocalcin (Gla-type osteocalcin in vitro enzyme immunoassay kit, Takara Bio Inc., UK) were measured using an enzyme-linked immunosorbent assay.

The intra and inter-assay precision coefficients of variation were 2.8% and 4.6% for FSH, 5% and 6.2% for LH, 9.9% and 11.8% for E2, 4.4% and 4.8% for testosterone, 4.3% and 7.8% for fT, 11% and 2.8% and 7% for A, 6.3% and 9.9% for DHEAS, 9.5% and 10.8% for 17-OHP, 5.2% and 5.8% for SHBG, and 1.6% and 6.1% for insulin, respectively. All results are expressed as means ± standard deviation.

### Statistical Analysis

The Shapiro-Wilk test was used to check the normality assumption of the data. Independent samples t-test and Mann-Whitney U tests were used to compare the differences of variables between the groups. Pearson and Spearman analysis were used to examine correlations, and a scatterplot matrix was also produced to display pairwise relationships between variables. To identify independent risk factors of PCOS, univariate and multivariate logistic regression analysis was used and odds ratios were calculated with their 95% confidence intervals. Statistically significant variables in univariate analysis were included in the multivariate logistic model and backward stepwise selection was performed at a stringency level of p<0.10 to determine the independent risk factors of PCOS. Two-sided p values <0.05 were considered statistically significant.

## RESULTS

The study and control groups were weight matched. Hormone levels and baseline characteristics of the groups are illustrated in Table 1.

The level of A was significantly high in the PCOS group. There was no statistically significant difference between the groups for age, BMI, DHEAS, FSH, SHBG, LH, fT, tT, and E2.

High levels of HOMA-IR, adiponectin, and osteocalcin were detected in the PCOS group. There was no significant difference between the two groups for TNF-α (Table 1). The cut-off value of HOMA-IR was accepted as 2.5^(12,13)^.

**Figure 1:**
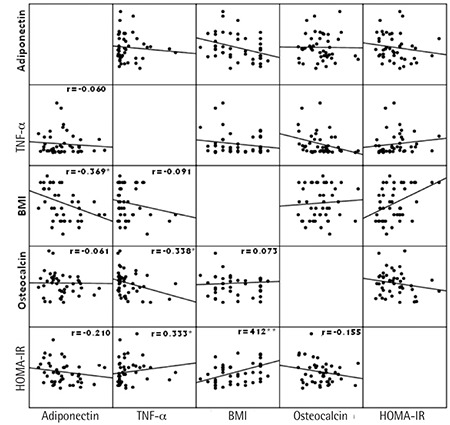
A scatterplot matrix displaying the relationship among body mass index, homeostasis model assessment-insulin resistance, tumor necrosis factor-alpha, adiponectin, and osteocalcin variables *p<0.05, **p<0.01 *HOMA-IR: Homeostasis model assessment-insulin resistance, BMI: Body mass index, TNF-α: Tumor necrosis factor-alpha*

We detected a strong positive correlation between adiponectin and osteocalcin in the control group. There was positive correlation between osteocalcin and BMI in addition to a negative correlation between osteocalcin and TNF-α in the PCOS group. We found a moderate positive correlation between BMI and HOMA-IR, a moderate negative correlation between TNF-α and osteocalcin, and a mild negative correlation between adiponectin and BMI (Table 2, Figure 1).

## DISCUSSION

Many of the symptoms appear to be quite heterogeneous, with marked differences in their prevalence and intensity among different groups of women with PCOS. IR was significantly high in the PCOS group. Some studies showed IR only in obese women with PCOS and others demonstrated IR in lean patients with PCOS. Of importance, the studies that failed to demonstrate IR in lean women with PCOS did, however, demonstrate elevated basal insulin levels compared with weight-matched controls without PCOS^(14)^.

The groups in our study were weight matched; therefore, the effect of adipose tissue on TNF-α and adiponectin was eliminated. We found higher levels of adiponectin in PCOS; however, some authors suggested that women with PCOS had lower adiponectin levels^(15)^. Conversely, an increment in plasma adiponectin was obtained by Frystyk et al.^(16)^ in type 1 DM. One way to interpret the present findings is to conclude that high adiponectin levels may be an early predictor of DM development. Unfortunately, 52.7% of patients in the PCOS group had IR. However, the finding can also be interpreted in the opposite way, as elevated adiponectin levels could represent a beneficial compensatory mechanism. Several markers of inflammation are increased in PCOS, which suggests that it is a state of chronic low grade inflammation. Keeping in mind the anti-inflammatory and anti-DM properties of adiponectin, one could hypothesize that increased adiponectin levels serve to protect patients at high risk of the harmful actions of pro-inflammatory and DM agents.

Increased levels of TNF-α were detected in PCOS; however, there was no statistical difference between the groups. Although Vural et al.^(17)^ could not illustrate higher TNF-α levels in PCOS, Xiong et al.^(18)^ suggested that patients with PCOS showed significantly higher serum TNF-α levels. The pathogenic impact of TNF-α in IR is underscored by the effect of the functional polymorphisms in the promoter regions of TNF-α, with different transcription rates^(19)^, or this situation may be related to the balance of anti-inflammatory and inflammatory agents that are secreted by bone and adipose tissue in PCOS.

More recently, evidence from animal studies suggested that the skeleton may exert an endocrine regulation of glucose metabolism. Lee et al.^(20)^ showed that mice lacking the gene that encodes osteocalcin had an abnormal amount of visceral fat and exhibited glucose intolerance, IR, and impaired insulin secretion compared with wild-type mice. Adami et al.^(21)^ could not illustrate a significant difference between a PCOS group and control group for osteocalcin, but they found normal androgen levels in their PCOS group; additionally, they did not examine patients for IR. In our study, osteocalcin was significantly increased in PCOS; moreover, there was a negative correlation between osteocalcin and TNF-α. Our study is in agreement with Diamanti-Kandarakis et al.^(22)^ who illustrated higher osteocalcin levels in PCOS.

There was no correlation between serum adiponectin and HOMA-IR. There was a moderate negative correlation between osteocalcin and TNF-α, in addition to a moderate positive correlation between BMI and HOMA-IR in PCOS. Adiponectin secretion is strongly related with IR rather than obesity, and a previous animal study showed that osteocalcin stimulated the expression of insulin in islets and of adiponectin in adipocytes with increased insulin secretion^(23)^. Perhaps increased osteocalcin levels contribute to high HOMA-IR by increased insulin secretion. Our groups were weight matched and the source of adiponectin was adipose tissue. This situation may explain why we did not detect a correlation between serum adiponectin levels and HOMA-IR.

TNF-α can be released from MNCs and hyperglycemia causes an increase in ROS generation from MNCs. Osteocalcin is defined in the literature with antidiabetic and anti-inflammatory properties, thus a plausible explanation of these events is that increased osteocalcin levels lead to a decrease in TNF-α.

### Study Limitations

Due to the relatively small sample size, our results display only weight-matched controls in PCOS. There is a need for further, larger scale studies including interactions between other genetic and environmental factors and the development of PCOS.

## CONCLUSION

Osteocalcin levels may have impact on adiponectin, TNF-α, and IR in PCOS. Therefore, osteocalcin may be responsible for PCOS heterogeneity.

## Figures and Tables

**Table 1:**
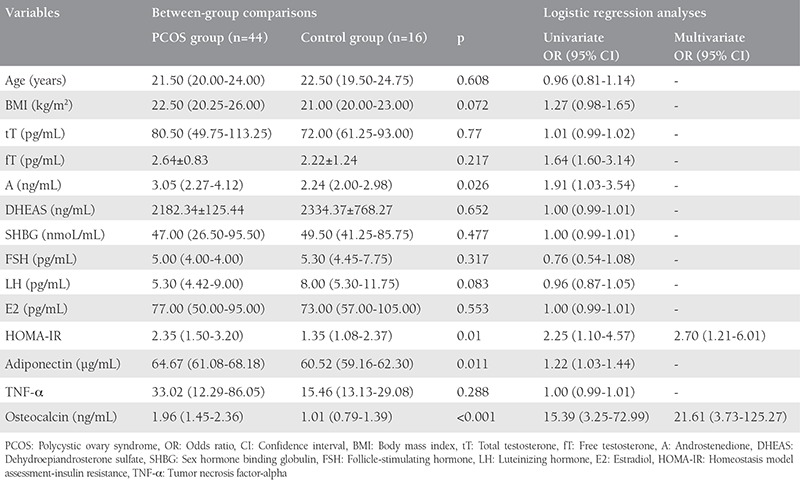
Hormonal levels and baseline characteristics of groups

**Table 2:**
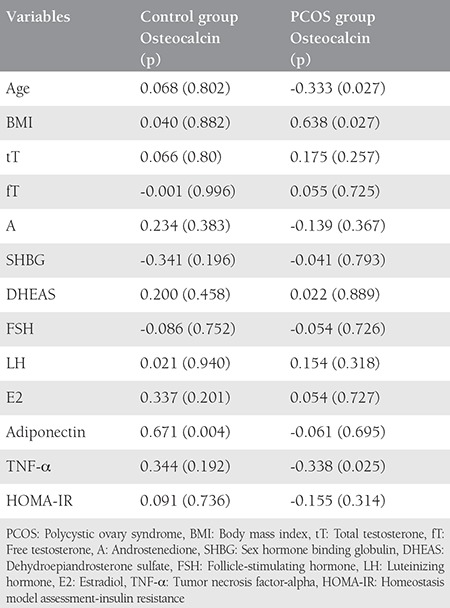
Correlation of osteocalcin level with hormonal levels, age, body mass index, homeostasis model assessment-insulin resistance, tumor necrosis factor-alpha, and adiponectin for both groups
